# A hemizygous p.R204Q mutation in the *ALAS2* gene underlies X-linked sideroblastic anemia in an adult Chinese Han man

**DOI:** 10.1186/s12920-021-00950-x

**Published:** 2021-04-15

**Authors:** Jinbo Huang, Meili Ge, Yingqi Shao, Min Wang, Peng Jin, Jiali Huo, Xingxin Li, Jing Zhang, Neng Nie, Yizhou Zheng

**Affiliations:** State Key Laboratory of Experimental Hematology, National Clinical Research Center for Blood Diseases, Institute of Hematology and Blood Diseases Hospital, Chinese Academy of Medical Science and Peking Union Medical College, 288 Nanjing Road, Tianjin, 300020 People’s Republic of China

**Keywords:** *ALAS2*, X-linked sideroblastic anemia, Hemizygous

## Abstract

**Background:**

X-linked sideroblastic anemia (XLSA) is the most common form of congenital sideroblastic anemia (CSA), and is associated with the mutations in the 5-aminolevulinate synthase 2 (*ALAS2*). The genetic basis of more than 40% of CSA cases remains unknown.

**Methods:**

A two-generation Chinese family with XLSA was studied by next-generation sequencing to identify the underlying CSA-related mutations.

**Results:**

In the study, we identified a missense *ALAS2* R204Q mutation in a hemizygous Chinese Han man and in his heterozygous daughter. The male proband presented clinical manifestations at 38 years old and had a good response to pyridoxine.

**Conclusions:**

XLSA, as a hereditary disease, can present clinical manifestations later in lives, for adult male patients with ringed sideroblasts and hypochromic anemia, it should be evaluated with gene analyses to exclude CSA.

**Supplementary Information:**

The online version contains supplementary material available at 10.1186/s12920-021-00950-x.

## Background

Congenital sideroblastic anemia (CSA) is a group of heterogeneous disorders characterized by hypochromic erythrocytes, increased serum iron and ferritin levels and bone marrow ringed sideroblasts [1, 2]. X-linked sideroblastic anemia (XLSA) is the most common form of CSA, and is attributed to the 5-aminolevulinate synthase (*ALAS2*) mutations [2, 3]. The human *ALAS2* gene is mapped on the X chromosome [4, 5], and encodes erythroid-specific *ALAS2*, which catalyzes the first and rate-limiting steps in the heme biosynthesis pathway in erythroid cells. According to the literatures since 2011 and Human Gene Mutation Data Base, over 100 missense mutations in the *ALAS2* gene have now been reported on XLSA patients.

Here, we report a missense mutation in the *ALAS2* gene in an adult male XLSA patient and his daughter.

## Materials and methods

### Patient

One patient with anemia was referred to the Institute of Hematology and Blood Diseases Hospital, CAMS and PUMC, Tianjin, China in December 2019, and was diagnosed as XLSA. His son and daughter also participated in the study. Written informed consents were obtained from the participant or the parents of the participants under the age of 18. The study was approved by the Ethics Committees of the Institute of Hematology, CAMS and PUMC (Ethics number: KT2017031-EC-1).

### Next-generation sequencing (NGS)

Genetic testing for hereditary blood and immune system diseases (including 700 genes) was performed in a panel by NGS. According to the standard procedure, a custom-crafted capture array (Nimblegen Sequence Capture array, Roche, USA) was used to capture all exons, splice sites, and the flanking intron sequences of all associated genes. SOAPsnp and Samtools pileup softwares were used to analyze gene mutation.

### Confirmation of candidate mutation by Sanger sequencing

The corresponding *ALAS2* gene region surrounding the R204Q mutation was amplified by PCR and sequenced by Sanger sequencing. PCR amplifications were performed in an ABI Veriti. Sequences were loaded on an ABI3500XL (Applied Biosystems). The sequence data files were analyzed using both Sequencing Analysis 5.2 and Variant Reporter v 1.1 software (Applied Biosystems). The primers for validating *ALAS2* R204Q mutation were as follows:

Forward primer: 5′ TTTTCATCCTCATATCTGCTCCTG 3′.

Reverse primer: 5′ TTGCTTGGTCTCCCATCCTTC3′.

## Results

**Fig. 1:**
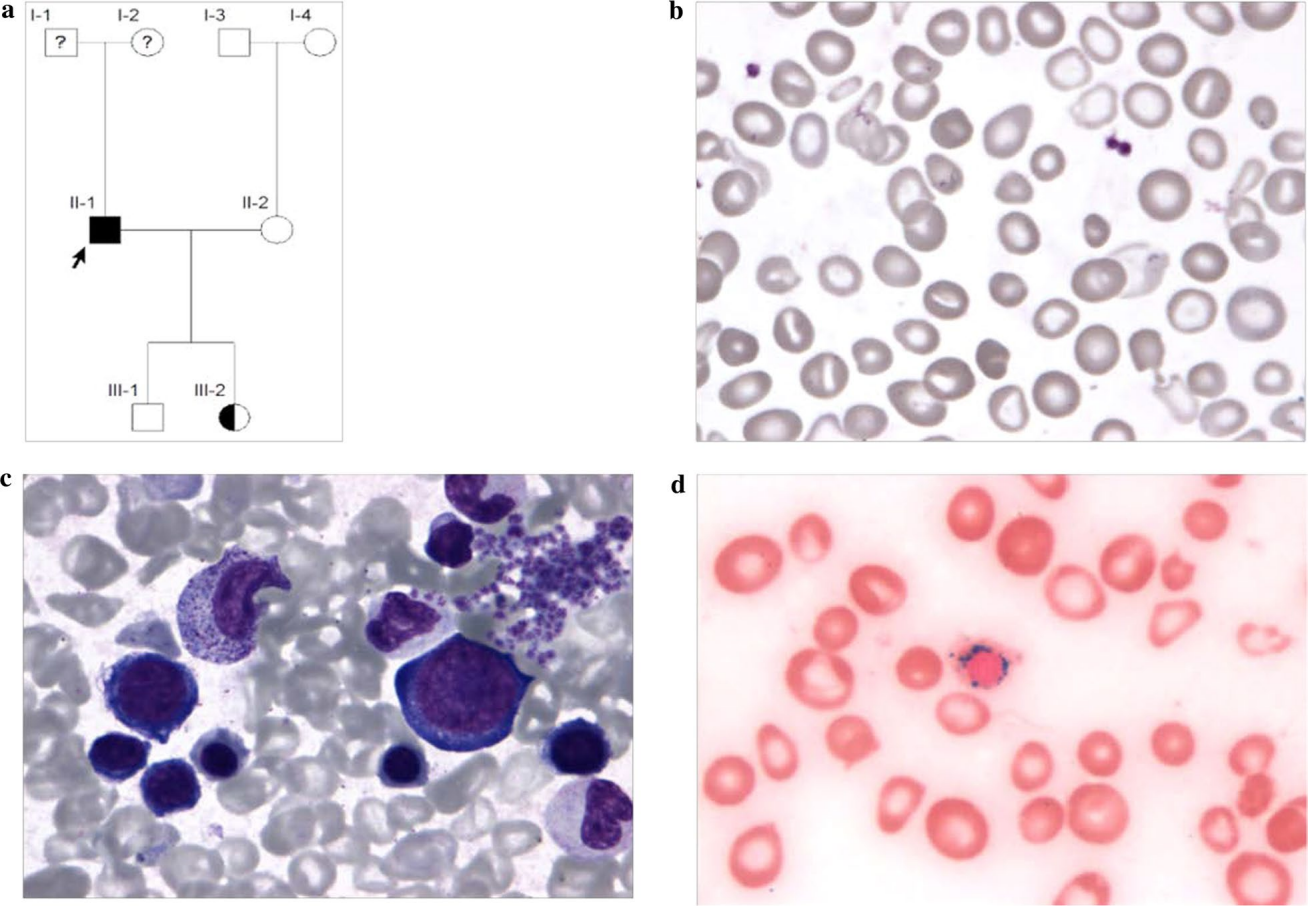
**a** Pedigree of a Chinese family with XLSA, the arrow indicated the proband. **b** The peripheral blood film from proband showed microcytic hypochromic, normocytic, and normochromic erythrocytes (HE stain, × 400). **c** The bone marrow film from proband showed mild dyserythropoietic change (HE stain, × 400). **d** The Prussian blue-stained specimens of bone marrow film from proband showed ringed sideroblasts with multiple perinuclear iron granules (HE stain, × 400)

Three individuals from a two-generation family were enrolled for the study, including the proband (II-1, 38 years), his healthy son (III-1, 12 years) and asymptomatic carrier daughter (III-2, 14 years) (Fig. 1a).

### Clinical findings

The patient, a 38-year-old Han Chinese male, lost contact with his biological parents and was brought up by foster parents. He was admitted to our hospital with a 2-month history of fatigue. He had normal stature and skin color, no hepatosplenomegaly. The red blood cell (RBC) counts were as follows: RBCs 4.53 × 10^12^/L (reference value: 4.0–5.5 × 10^12^/L), hemoglobin (Hb) 91 g/L (reference value: 120–160 g/L), mean corpuscular volume (MCV) 74.2fL (reference value: 80–100 fL), mean corpuscular hemoglobin concentration (MCHC) 271 g/L (reference value: 320–360 g/L), red blood cell distribution width (RDW) 31.8% (reference value: 11.1–14.1%), Reticulocyte (RET) rate 2.87% (reference value: 0.5–1.5%). The peripheral blood film was dimorphic, including microcytic hypochromic, normocytic, and normochromic erythrocytes (Fig. 1b). Ferritin was 625.9 ng/mL (reference range: 23.9–336.2 ng/mL). Thalassemia gene test was negative. Bone marrow analysis revealed marked erythroid hyperplasia with mild dyserythropoietic changes (Fig. 1c). The ringed sideroblasts with multiple perinuclear iron granules constituted over 40% of total erythroblasts in the Prussian blue-stained specimens (Fig. 1d). The RBC counts of the patient’s asymptomatic carrier daughter were as follows: RBCs 4.80 × 10^12^/L, Hb 127 g/L, MCV 82.0 fL, MCHC 321 g/L, RDW 14.1%.

Oral pyridoxine (60 mg, three times a day) was prescribed to the XLSA patient with ALAS2 p.R204Q mutation, Hb rose to 149 g/dL after 3 months course and remained normal at the 9-month follow-up. However, erythrocytes still showed microscopic and hypochromic, with little improvement. The patient didn’t receive iron chelation therapy. Ferritin was 885.6 ng/mL after 6 months, and 957.9 ng/mL after 9 months.

### Genetic findings

**Fig. 2:**
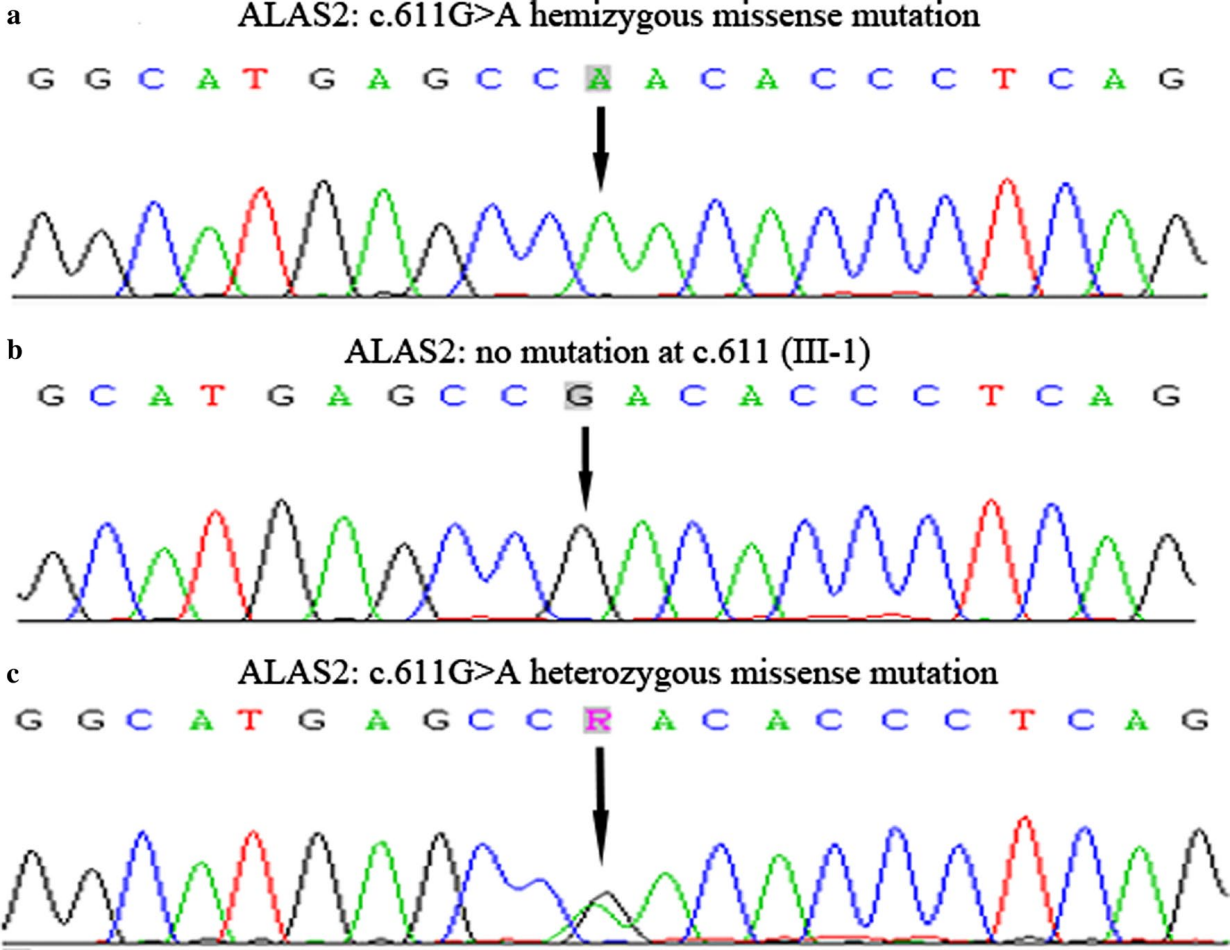
**a** A hemizygous mutation (c.611G>A; p.R204Q) in the proband with XLSA. **b** The proband’ son had no mutation at C.611. **c** The proband’ daughter harbored a heterozygous mutation (c.611G>A; p.R204Q)

We detected a missense mutation for a G to A transition in exon 5 of the *ALAS2* gene, which caused a arginine (Arg) to glutamine (Gln) substitution at amino acid position 204 (NM_000032.5: c.611G>A; p.R204Q) in the proband and his daughter (Fig. 2a, c), which located at the domains important for pyridoxal phosphate co-factor binding [6, 7]. His son (III-1) was normal (Fig. 2b). Different predictive tools were used to determine the significance of the missense mutation: Polyphen-2 (the mutation was predicted to be “possibly damaging” with a score of 0.673) (http://genetics.bwh.harvard.edu/ggi/pph2/69eced31378726308ea4c615c8598d621e117817/6950834.html), PROVEAN (the mutation was predicted to be “deleterious” with a score of -2.72) and SIFT(the mutation was predicted to be “tolerated” with a score of 0.497) (http://provean.jcvi.org/protein_batch_view_table.php?jobid=798947818062689). The results suggested that this substitution had a moderate probability for affecting the protein function.

## Discussion

We found an ALAS2 missense mutation (p.Arg204Gln) in a hemizygous adult man and in his heterozygous daughter. The mutation has already been reported [8, 9]. The reported male patient presented clinical manifestations at 38 years old, with ringed sideroblasts and microscopic hypochromic anemia. In adults, ringed sideroblasts are commonly associated with MDS, and often result from splicing factor 3b, subunit 1 (*SF3B1*) mutation [10]. However, because mild, life-long anemia may not be detected because of lack of symptoms, asymptomatic subjects are discovered through routine blood examination or family surveys, some XLSA patients can present clinical manifestations later in their lives. The oldest reported case was an 81-year-old male who suffered with chronic renal failure, pyridoxine deficiency caused by maintenance hemodialysis therapy resulted in obvious anemia and uncovered occult inherited enzymatic deficiencies [11]. Recently, another late-onset XLSA also had been reported [12]. A few reports had described the misdiagnosis of adult patients with XLSA as MDS with refractory anemia with ringed sideroblasts (MDS-RARS) [13]. In addition, women with XLSA nearly always have normocytic or macrocytic anemia that has to be distinguished from MDS-RARS, they develop symptomatic XLSA later in life due to acquired skewing of X chromosome inactivation in hematopoietic cells leading to predominance of an active X which bears an ALAS2 mutation. XLSA and MDS-RARS have the shared morphological features, such as the dyserythropoiesis and ringed sideroblasts, so it may be required to exclude XLSA by the identification of the ALAS2 mutation.

*ALAS2* catalyzes the first step of heme biosynthetic pathway by condensing glycine and succinyl-CoA to form delta-aminolevulinic acid in the presence of a pyridoxal 5′-phosphate, which is the metabolite of vitamin B6 [14]. In the process of ALA synthesis, PLP is a cofactor reversibly bound to Lysine 391 and its binding is absolutely required for enzymatic activity [15]. All reported *ALAS2* mutations in male XLSA patients were missense mutations, mostly located at the domains important for pyridoxal phosphate co-factor binding or catalysis [6, 7]. Pyridoxine can enhance the activity of *ALAS2* enzyme, more than half of XLSA patients have response to pyridoxine [1]. Our reported ALAS2 mutation (p.Arg204Gln) is located on the surface of the ALAS2 3D structure some distance from the PLP moiety that is buried in the middle of the structure [6]. We can only speculate that the presence of Gln at the R204 location destabilizes the enzyme's structure but the binding of the PLP cofactor stabilizes the mutant structure somewhat. Consistent with this line of reasoning, others have shown that this mutant enzyme's activity was only 15.1% of that of the normal control but could be increased up to 34.5% with the addition of pyridoxine [8]. This finding was confirmed by the good response to pyridoxine treatment observed for the proband in our study.

CSA patients are prone to iron overload, whether pyridoxine-responsive or not, regardless of red blood cell transfusions. Iron overload is partly attributed to reduced hepcidin level secondary to ineffective erythropoiesis which promotes intestinal iron absorption [16]. Our reported XLSA patient had no red blood cell transfusion and had a good response to pyridoxine, the ferritin still increased gradually, iron depletion by phlebotomy should be initiated if serum ferritin is over 1000 mcg/L.

## Conclusions

In summary, we identified an X-linked *ALAS2* R204Q mutation in a hemizygous Chinese Han man and in his heterozygous daughter. The male patient showed clinical presentation at the adult age. It should be evaluated with gene analyses to exclude CSA for adult male patients with ringed sideroblasts and microscopic hypochromic anemia.

## Supplementary Information

**Additional file1:** Data title: the ALAS2 sequence of the proband, his daughter and son by Sanger sequencing

## Data Availability

The sequence of *ALAS2* gene tested by Sanger sequencing used and/or analyzed during the current study are available in the Additional file 1. All other data generated or analysed in this study were included in this article.

## Abbreviations

ALAS2: 5-Aminolevulinate synthase
CSA: Congenital sideroblastic anemias
FL: Flt3 ligand
IL-3: Interleukin-3
Hb: Hemoglobin
MCHC: Mean corpuscular hemoglobin concentration
MCV: Mean corpuscular volume
MDS: Myelodysplastic syndrome
MDS-RARS: MDS with refractory anemia with ringed sideroblasts
TPO: Thrombopoietin
RBCs: Red blood cells
RET: Reticulocyte
SF3B1: Splicing factor 3b, subunit 1
SCF: Stem cell factor
XLSA: X-linked sideroblastic anemia

## Declarations

### Methods

All methods used during the current study were carried out in accordance with relevant guidelines and regulations.
