# Supplementary material for: A hemizygous p.R204Q mutation in the ALAS2 gene underlies X-linked sideroblastic anemia in an adult Chinese Han man
Source: BMC Med Genomics. 2021 Apr 15;14:107. doi: 10.1186/s12920-021-00950-x (PMC8048311; doi:10.1186/s12920-021-00950-x)

**SUPPLEMENTARY INFORMATION**

# A hemizygous p.R204Q mutation in the ALAS2 gene underlies X-linked sideroblastic anemia in an adult Chinese Han man

Jinbo Huang[1], Meili Ge[1*], Yingqi Shao[1], Min Wang[1], Peng Jin[1], Jiali Huo[1], Xingxin Li[1], Jing Zhang[1], Neng Nie[1], and Yizhou Zheng[1]

[1]State Key Laboratory of Experimental Hematology, National Clinical Research Center for Blood Diseases, Institute of Hematology & Blood Diseases Hospital, Chinese Academy of Medical Science & Peking Union Medical College, 288 Nanjing Road, Tianjin 300020, P.R.CHINA

**Supplementary file**: the *ALAS2* sequence of the proband, his daughter and son

The corresponding *ALAS2* gene region surrounding the R204Q mutation was amplified by PCR and sequenced by Sanger sequencing in proband and his son and daughter. The raw ALAS2 sequence of the proband, his daughter and son by Sanger sequencing was provided as following, which showed the location of *ALAS2* mutation.

1. **The ALAS2 sequence of the proband's daughter by Sanger sequencing.**

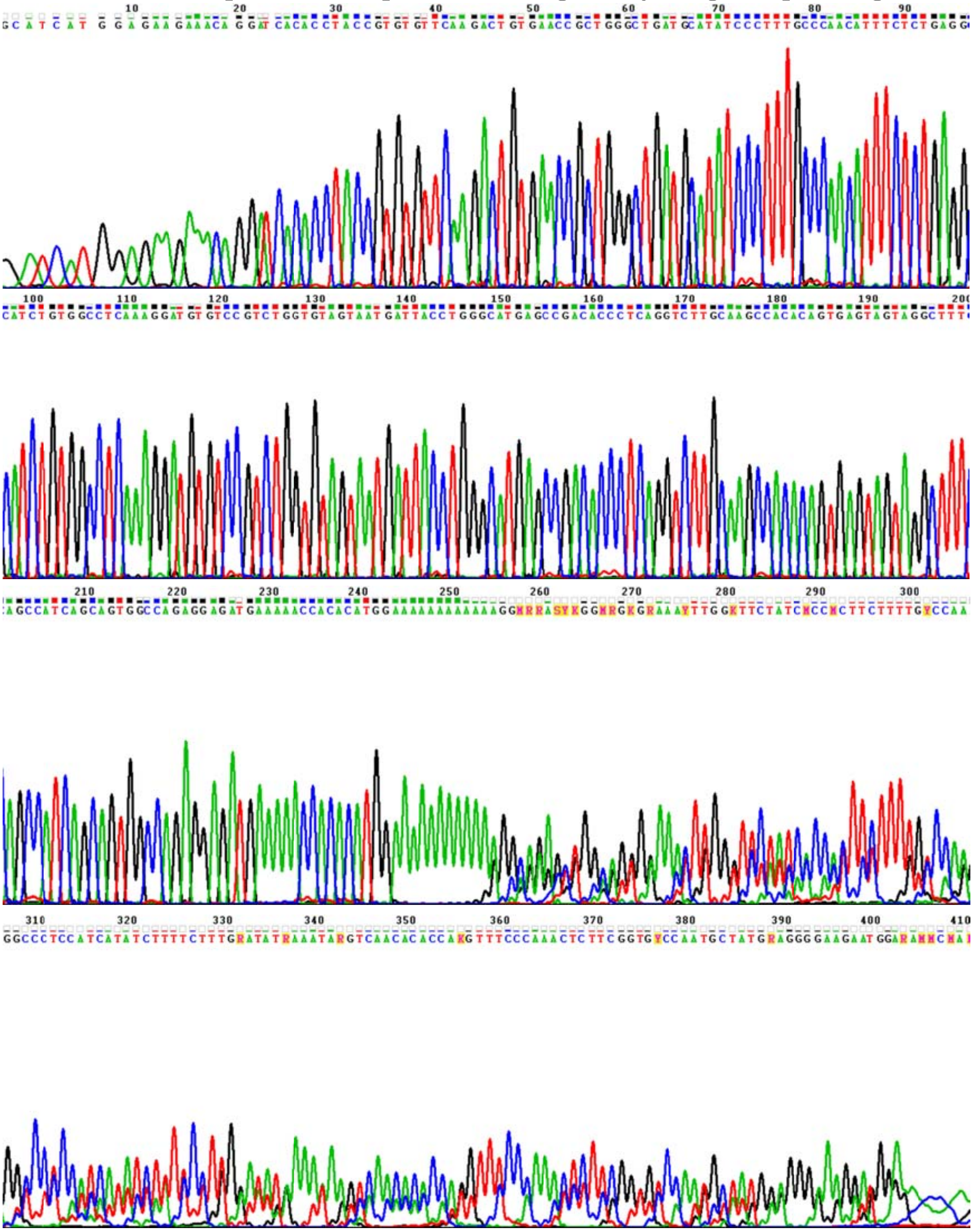

**2. The ALAS2 sequence of the proband by Sanger sequencing.**

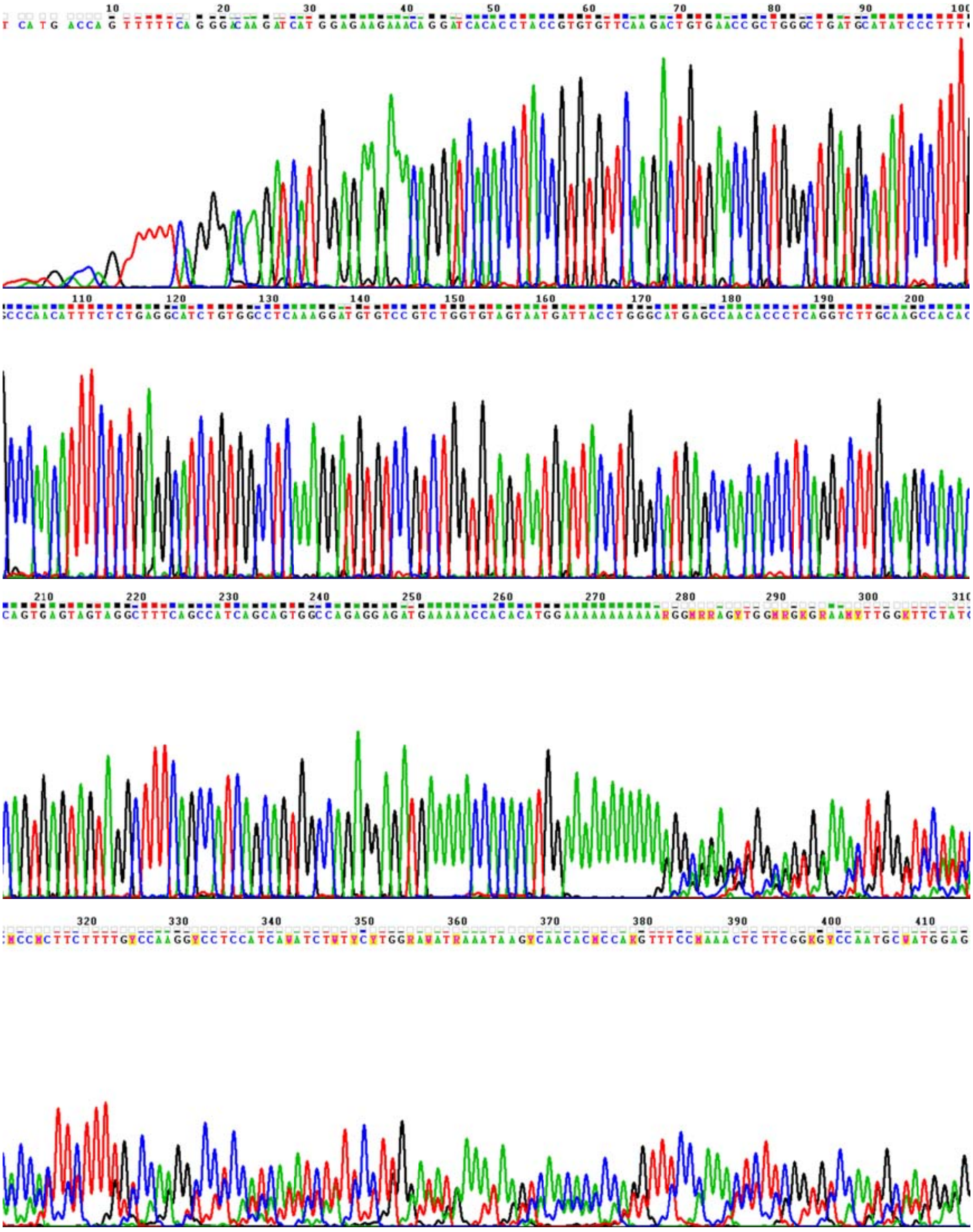

**3. The ALAS2 sequence of the proband's son by Sanger sequencing.**

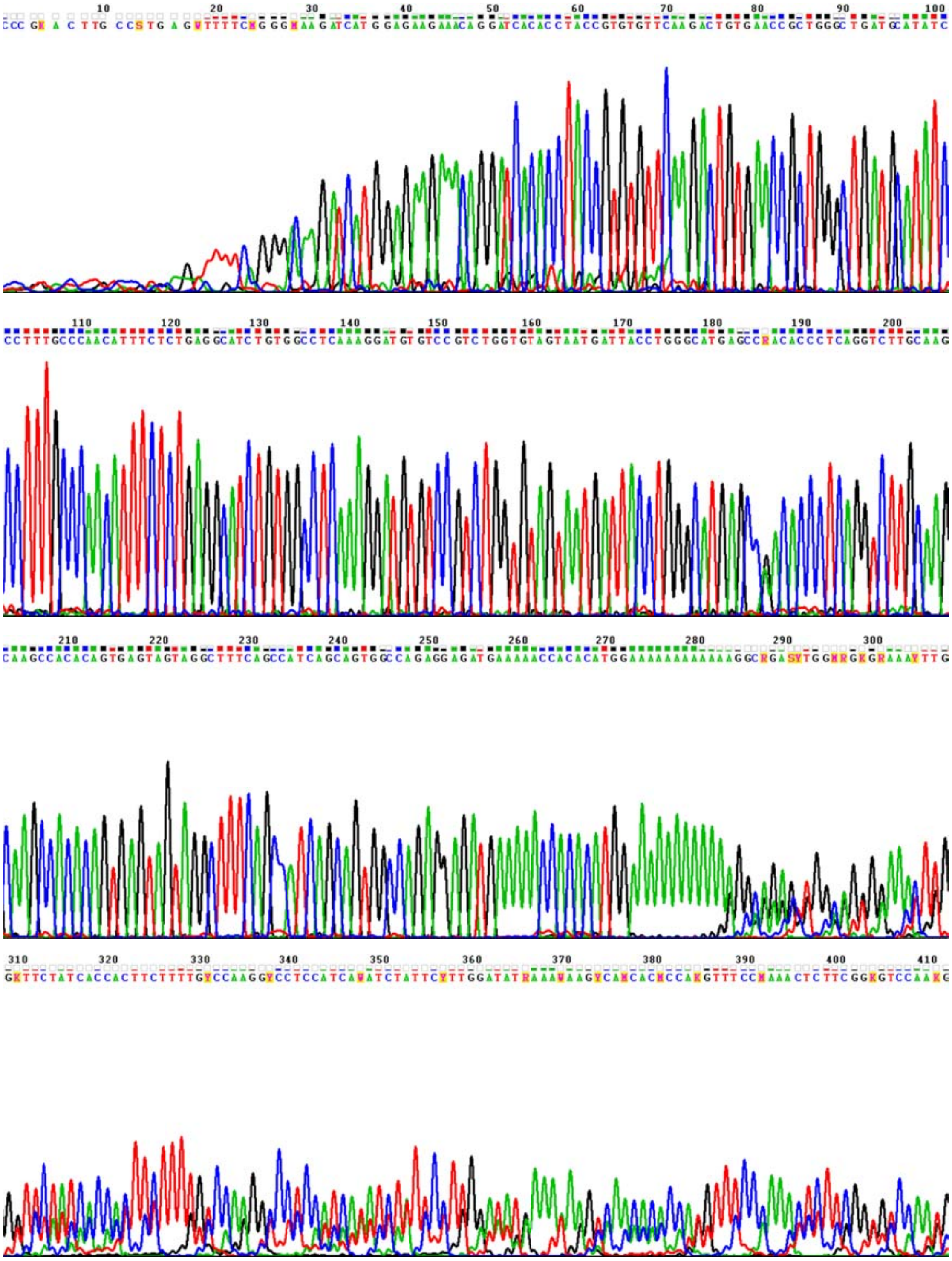

Supplement: Supplementary file 1 — Additional file1: Data title: the ALAS2 sequence of the proband, his daughter and son by Sanger sequencing [file 12920_2021_950_MOESM1_ESM.pdf]
